# Cartas al editor

**Published:** 2020-06-30

**Authors:** Fabio Samir Vargas-Cely, Andrés F. Zea-Vera

**Affiliations:** 1 Médico interno, Universidad del Valle, Hospital Universitario del Valle, Cali, Colombia; Departamento de Microbiología, Universidad del Valle, Cali, Colombia Universidad del Valle Universidad del Valle Hospital Universitario del Valle Cali Colombia; 2 Departamento de Microbiología, Universidad del Valle, Cali, Colombia; Inmunología Clínica, Hospital Universitario del Valle, Cali, Colombia Universidad del Valle Departamento de Microbiología Universidad del Valle Cali Colombia

Cali, 6 de marzo de 2020

Señores Comité Editorial

Revista *Biomédica*

Bogotá

Estimados señores:

Recientemente leímos un reporte de caso publicado en su revista llamado “Abscesos cerebrales por *Nocardia* spp. en una paciente inmunocompetente”, de Trujillo, *et al.*^1^, sobre el que quisiéramos hacer algunas acotaciones.

Es usual encontrar en la práctica clínica y en las publicaciones científicas conclusiones que atribuyen un estado de “inmunocompetencia” a los individuos con base únicamente en la exclusión del HIV o, en algunos casos menos frecuentes, de otras causas de inmunodeficiencia secundaria ^2^^,^^3^, tal como ocurre en el reporte de caso en mención. En este sentido, es necesario aclarar que el estado de inmunocompetencia trasciende la mera exclusión de las condiciones patológicas asociadas e implica, por lo menos, una evaluación objetiva básica de las variables cuantitativas y cualitativas de la respuesta inmunológica mediante herramientas disponibles en la práctica clínica, la cual debe responder de forma individualizada al perfil de infección del paciente en aquellos casos relacionados con infecciones atípicas, recurrentes o de difícil manejo ^4^.

Los autores mencionan que: “[…] La aparición de nocardiosis en un paciente inmunocompetente es rara, por lo que se debe descartar la inmunosupresión por cualquier causa […]”, sin embargo, solo mencionan la exclusión de las principales causas de inmunodeficiencia secundaria, sin informar si habían evaluado parámetros inmunológicos accesibles en la práctica clínica, como la cuantificación de linfocitos T, B, células NK, niveles de inmunoglobulinas séricas (IgG, IgA, IgM o IgE), o la función de los fagocitos (reducción de dihidrorrodamina, DHR 123), y si habían hecho pruebas genéticas para encontrar mutaciones en genes fundamentales para la respuesta inmunológica (paneles y secuenciación exómica). En caso de ser normales, dichos exámenes paraclínicos pueden precisar una definición de “inmunocompetencia” o demostrar alguna alteración indicativa de la presencia de un trastorno intrínseco primario de la respuesta inmunitaria, o de inmunodeficiencia primaria, que a pesar de ser más prevalentes en la niñez, también pueden manifestarse clínicamente en la adultez o ser diagnosticadas tardíamente ^5^.

Con relación a la infección del caso que comentamos, además de ser atípica, fue agresiva y comprometió tejidos blandos, el pulmón y el sistema nervioso central, lo que configura una situación de alerta por un estado de inmunodeficiencia, incluso primaria, como se ha reportado en algunas series de nocardiosis ^6^. Se podrían considerar dos probables inmunodeficiencias primarias que podrían explicar el cuadro: en primer lugar, la susceptibilidad (sic) mendeliana a las micobacterias asociada con defectos intrínsecos de las vías de la IL-12 e IL-23 dependientes de IFN-gamma, fundamentales para el control de infecciones por micobacterias y *Nocardia* spp. ^7^; y, en segundo lugar, la enfermedad granulomatosa crónica, más probable en este caso dado el fenotipo clínico de la paciente.

Esta enfermedad es una inmunodeficiencia primaria en la que las mutaciones en alguna de las cinco subunidades de la NADPH oxidasa de los fagocitos resultan en la disminución de la capacidad de estallido respiratorio en estas células, defecto que se suma a la presencia de factores de virulencia típicos de *Nocardia* spp., como la expresión de la catalasa y de la superóxido dismutasa, que aumenta la capacidad de evasión de los mecanismos de defensa mediados por el estallido respiratorio ^8^ (ya bastante disminuidos en la enfermedad granulomatosa crónica), así como la sensibilidad a otros microorganismos como *Aspergillus* spp., *Serratia marcescens* y *Salmonella* no tifoidea ^9^.

En la práctica clínica sugerimos solicitar un test de estallido respiratorio de fagocitos con DHR 123, así como un panel genético en busca de mutaciones en este complejo enzimático, lo que puede demostrar defectos funcionales en los fagocitos asociados con mutaciones genéticas puntuales que explicarían un cuadro secundario a una probable enfermedad granulomatosa crónica, como ha ocurrido en casos en los que esta se ha diagnosticado en la adultez, con características clínicas y microbiológicas casi idénticas a las presentadas en el caso en mención ^10^.

Dado lo anterior, sugerimos no clasificar a los pacientes como inmunocompetentes si este tipo de evaluaciones no se han realizado y, en caso de haberlo hecho, mencionarlo en los manuscritos.
