# Supplementary material for: Cartas al editor
Source: Biomedica. 2020 Jun 30;40(2):412–4. [Article in Spanish] (PMC7505511)
Supplement: Related texts

# Reply: Cartas al editor

**Authors:** Danilo Eduardo Trujillo-González

**Affiliations:** 1 Residente de Medicina Interna, Universidad del Rosario, Fundación Cardio- Infantil, Instituto de Cardiología, Bogotá, D.C., Colombia Universidad del Rosario Universidad del Rosario Fundación Cardio- Infantil Instituto de Cardiología BogotáD.C Colombia

Bogotá, D.C., 6 de mayo de 2020 Señores

Comité Editorial Revista Biomédica La ciudad

Estimados señores:

Ante todo, agradecemos los comentarios de Vargas, *et al.,* a nuestra publicación “Abscesos cerebrales por *Nocardia* spp. en una paciente inmunocompetente” (Biomédica. 2020;40:27-33. https://doi.org/10.7705/biomedica.4925). Entre los exámenes especializados de hematología e infectología que se le hicieron a la paciente del caso que presentamos, se midió el nivel de inmunoglobulinas séricas (inmunoglobulinas G, M y E), el cual fue normal. Los demás exámenes paraclínicos reseñados por los autores de la carta (conteo de células *natural killer*, conteo diferencial de linfocitos T, evaluación de competencia de células fagocíticas y estudios genéticos) no se hicieron.

La prevalencia de la enfermedad granulomatosa crónica en población adulta no se conoce ^1^ y, si bien se ha descrito su asociación con la nocardiosis en la práctica clínica, la pertinencia de hacer dichos estudios en un paciente hospitalizado no es clara ^2^.

Hasta el momento no hay una directriz inequívoca sobre la necesidad de hacer estudios inmunogenéticos, como tampoco una guía que nos oriente en torno a estudios adicionales en casos similares de pacientes adultos sin otras manifestaciones de la enfermedad ^3^, por lo que, dada la adecuada respuesta de la paciente al tratamiento durante el seguimiento, el Servicio de Infectología no consideró la necesidad de hacerlos.

Les agradecemos la recomendación y en futuras oportunidades se tendrá en cuenta la pertinencia de la evaluación inmunológica completa.

## References

[1] van den Berg JM, van Koppen E, Ahlin A, Belohradsky BH, Bernatowska E, Corbeel L (2009). Chronic granulomatous disease: The European experience. PLoS One.

[2] Roos D (2016). Chronic granulomatous disease. Br Med Bull.

[3] Mortaz E, Azempour E, Mansouri D, Tabarsi P, Ghazi M, Koenderman L (2019). Common infections and target organs associated with chronic granulomatous disease in Iran.
